# Coverage of Quality Maternal and Newborn Healthcare Services in India: Examining Dropouts, Disparity and Determinants

**DOI:** 10.5334/aogh.3586

**Published:** 2022-05-26

**Authors:** Lucky Singh, Ritam Dubey, Prashant Kumar Singh, Saritha Nair, Rajesh Kumar Rai, M. Vishnu Vardhana Rao, Shalini Singh

**Affiliations:** 1 ICMR National Institute of Medical Statistics, Ansari Nagar, New Delhi 110029, IN; 2 Division of Preventive Oncology and Population Health, ICMR National Institute of Cancer Prevention and Research, I – 7 Near City Centre Metro Station, Sector 39, Noida, Uttar Pradesh 201301, IN; 3 Society for Health and Demographic Surveillance, Suri, West Bengal, India 731101, IN; 4 ICMR National Institute of Cancer Prevention and Research, I – 7, Sector 39, Noida, Uttar Pradesh 201301, IN

**Keywords:** maternal and child health, quality of care, continuum of care, health equity; India

## Abstract

**Background:**

Abundant research studies has recorded availability, accessibility and quality of antenatal care and safe delivery in India but comparatively less information is known for postnatal care and furthermore limited attempts at capturing the whole spectrum of obstetric and newborn health services. Assessing discontinuity in maternal and child health service utilization provides us holistic information about existing health inequities and barriers in service provision.

**Objective:**

Current study evaluated the coverage of quality antenatal care (QANC), delivery care (QDC) and postnatal care (QPNC) in India as a part of a single continuum accounting for significant regional and sub-regional disparities.

**Methods:**

This study analyzed nationally representative data obtained from NFHS-4 (2015–16). Included in the data, were 190 898 Indian women who had a recent birth in last five years. Coverage of QANC, QDC and QPNC was examined at the national, state and district level. Bivariate association of key sociodemographic variables with coverage of services was assessed during chi-squared analysis. Multilevel logistic regression analysis examined correlates associated with coverage of services. The output was presented using odds ratios (OR) with 95% CI.

**Findings:**

About 23.5% women utilized QANC out of which 92.9% opted for QDC and 35.1% of newborns received QPNC. About 400 and 471 districts out of 640 had less than 30% coverage of QANC and QPNC, respectively. Women residing in rural regions of Bihar and Northeastern states were found with less than 10% coverage of QANC. Regression analysis shows that women with more than 12 years of education and belonging to richest households had increased odds of availing QANC (OR 1.95; 95%CI: 1.84–2.06) and QDC (OR: 2.86; 95%CI: 2.27–3.60), respectively.

**Conclusion:**

Focused interventions targeting the delivery of quality services especially ANC and PNC among newborns are imperative to achieve SDG-3 goals to achieve improvement in maternal and newborn health.

## Introduction

Prioritization of maternal and newborn healthcare in the Sustainable Development Goals (SDG) is yet to translate to improvement in maternal and newborn mortality statistics in the Sub-Saharan African and Southeast Asian regions [1]. India shares burden of about 63 percent of total neonatal deaths in South Asia [2] and is the second largest contributor to the global tally of maternal deaths [3]. More than a quarter of neonatal deaths have been noted to occur within first 24 hours in the country [4] and hence the care received during this duration is an essential component of the continuum of care.

Continuum of care (CoC) in relation to maternal and newborn health calls for viewing provision of key services across the pregnancy, childbirth and postpartum period as a single unit with the objective of providing safe and fulfilling pregnancy and delivery experience in addition to providing appropriate health services to newborns [5]. Assessing the coverage and quality aspects of MCH services as part of the continuum is imperative to gain a fair idea about service provision gaps and discontinuity in utilization. Despite of having appropriate maternal and child healthcare policies in place the country faces significant regional inequities in reproductive health [6]. These inequities strongly determines the healthcare service utilization [7,8] and accordingly defines the prioritization of health interventions such as categorization done by the Government of India of all the states into high focus and non-high focus states. Therefore, it is imperative to identify the need gaps existing for each of the services provided in the continuum to inform better policy interventions to aid in reducing overall maternal and newborn mortality rates. The present study assesses coverage of quality of healthcare along the continuum of care at the national, regional and sub-regional (districts) levels in order to derive policy implications that can be translated to efficient and practical interventions at the macro and primary administrative units of the country.

## Methods

### Study design and sample

Secondary analysis of the nationally representative data from the fourth round of National Health and Family Survey (NFHS-4, 2015–16) was conducted by the present study. Information about antenatal to postnatal care was considered from 190 898 eligible women aged 15–49 years who had their most recent birth in the five years preceding the survey date.

### Sample procedure followed by NFHS-4 (2015–16)

The survey used two stage-stratified sampling design, with villages in rural areas and Census Enumeration Blocks in the urban areas as the Primary Sampling Units (PSUs). A total of 28 586 PSUs were selected across India out of which survey was completed in 28 522.The survey was done on 723 875 eligible women (aged 15–49). Survey obtained nationwide representative clinical, anthropometric, and biochemical information from four questionnaires covering 601 509 households across 7 union territories, 29 states and 640 districts with a response rate of 98%. Further information about the survey is available in the published report [9].

### Measures

Outcome variable comprised of quality of care received throughout the tenure of pregnancy, during delivery and to the newborns. Quality Antenatal Care (QANC) considered the following dimensions,

- Skilled: Care received from auxiliary nurse midwives, lady health visitors, doctors, nurses or midwives.
- Timely: Completion of first ANC visit and registration of the pregnancy within first trimester of the pregnancy.
- Sufficient: At least four ANC visits to be completed during the period of pregnancy.
- Appropriate: Indicator summarizing the procedures and processes of care provided during at least one antenatal care visit.

This study has considered the procedures as mentioned below,

(1) weight measurement, (2) blood pressure measurement, (3) urine testing, (4) blood sample taken to test for possible morbidities such as anaemia, parasite infestations or infectious diseases, (5) at least two tetanus vaccinations, (6) iron and folic acid tablets were consumed for at least 100 days (7) abdominal examination, and (8) whether counseling was given regarding specific symptoms of pregnancy complications and information about the place to approach for, if any complications arise. Seven out of eight of these procedures were considered for ANC service to be considered as appropriate in accordance with similar studies [10,11]. Inadequate ANC was ascertained when the dimensions did not strictly meet the criteria set out by each of the above-mentioned dimensions (for instance, ANC received from unskilled health personnel) and a woman was considered to have received no ANC when the services provided under each dimension were either absent or did not meet even one criteria.

Quality delivery care (QDC) accounted for skilled attendance at birth as per the guidelines released by the Government of India [12]. Literature has noted first 24 hours after delivery associated with about one-third of all the neonatal deaths in the country [13]. The study considered postpartum care received to be quality postnatal care (QPNC) when services were received by skilled health personnel within 24 hours of birth, either at home or at the institution.

Factors were categorized at the individual, household and contextual levels to include a holistic spectrum of environmental and population characteristics in the analysis in accordance with the Anderson’s model of seeking healthcare [14]. Demographic and socio-economic characteristics such as woman’s age at childbirth, parity, education, household wealth, community poverty and illiteracy were considered under predisposing factors. Women’s age at childbirth, educational status, parity was included at the individual level. Gender composition of living children (considered in the study as a woman having no sons or at least one son among all her living children in a household) was included to assess the association between son preference and utilization of maternal and newborn healthcare services [15]. Under enabling factors, variables such as place of current residence and whether the woman resides in a high focus state were taken. These factors were further classified at the individual, household and contextual level in order to evaluate individual, community and regional influences of healthcare service utilization across the continuum. Women’s age at childbirth, educational status, parity was included at the individual level. Wealth quintile, caste and religion were included to consider the variables affecting health outcomes at the household level [16]. At the contextual level, socioeconomic factors such as proportion of illiterate and poor under 4 categories (0–25%, 25–50%, 50–75% and 75–100%) at the PSU level. Additionally, current place of residence (rural/urban) and region of residence in terms of high and non-high focus states were considered in line with recent literature [17,18]. “High focus states” comprises of Jharkhand, Bihar, Chhattisgarh, Orissa, Madhya Pradesh, Rajasthan, Assam, Uttarakhand and Uttar Pradesh and non-High focus states included rest of the states [19].

### Statistical Analysis

Coverage of QANC, QDC and QPNC were examined at the national, state and district levels and dropouts along the CoC were calculated and presented as a flow chart. All the three components were mapped across 640 districts of India using ArcGIS. The association of key socioeconomic and demographic variables with QANC, QDC and QPNC was assessed using chi-square bivariate analysis and were presented as percentages with their 95% confidence intervals. Following, multilevel logistic regression analysis was used in consideration of the hierarchical nature of data to examine correlates associated with utilization of QANC, QDC and QPNC while considering range of sociodemographic characteristics of population. Study used Stata version 13 [20] to analyze the data for the present study and “svy” suite in-built with the software used to apply appropriate sample weighting.

### Ethics and Consent

Patients and/or the participants were not involved in the development of research question, design, conduction, reporting, or dissemination plans of this research as this study involves secondary research of the data collected in the NFHS-4 survey. The information collected in the survey was collated so that the personal identifiers were not disclosed. The dataset used in this study is also available in the public domain from the DHS Program webpage (https://dhsprogram.com/data/).

## Results

### Sample characteristics

Supplementary Table 1 describes the distribution of total sample of 190 898 women considered in the present study. Majority of the women included in the study belonged to the age range of 20 to 24 years (39.5%) and received no formal education (29.1%). Fifteen point one percent belonged to the richest quintile. More women resided in the high focus states (58.9%), in rural areas (74.9%) and belonged to the central region (27.7%) of the country. Majorities (72.5%) of the women were Hindus and belonged to OBC (38.8%) social class.

### Dropouts in quality ANC, delivery and PNC along the continuum of care

**Figure 1:**
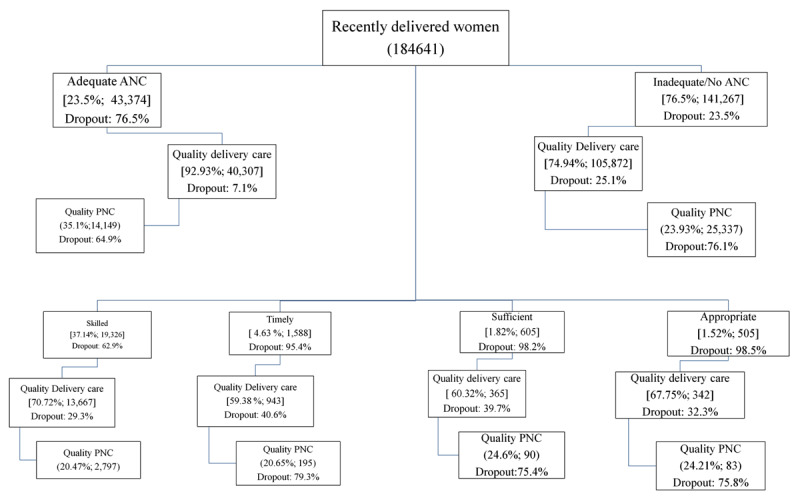
Utilization of quality maternal and newborn healthcare services and its components along the continuum of care at the national level (2015–16).

**Figure 2:**
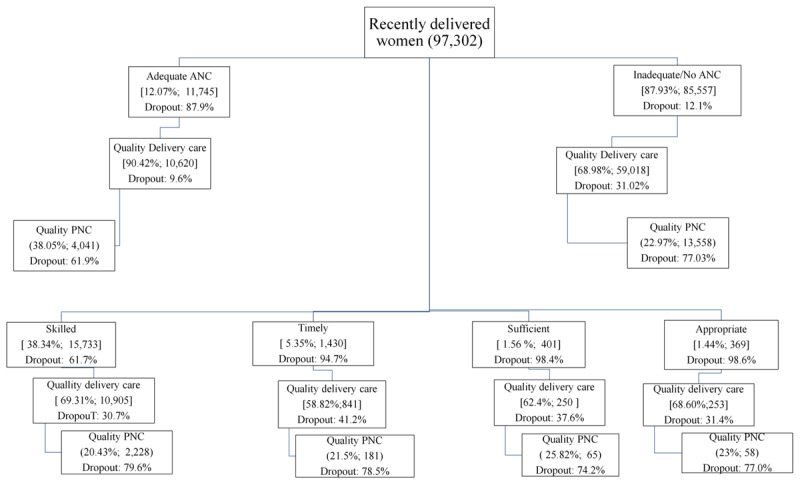
Utilization of quality maternal and newborn healthcare services and its components along the continuum of care in rural India (2015–16).

**Figure 3:**
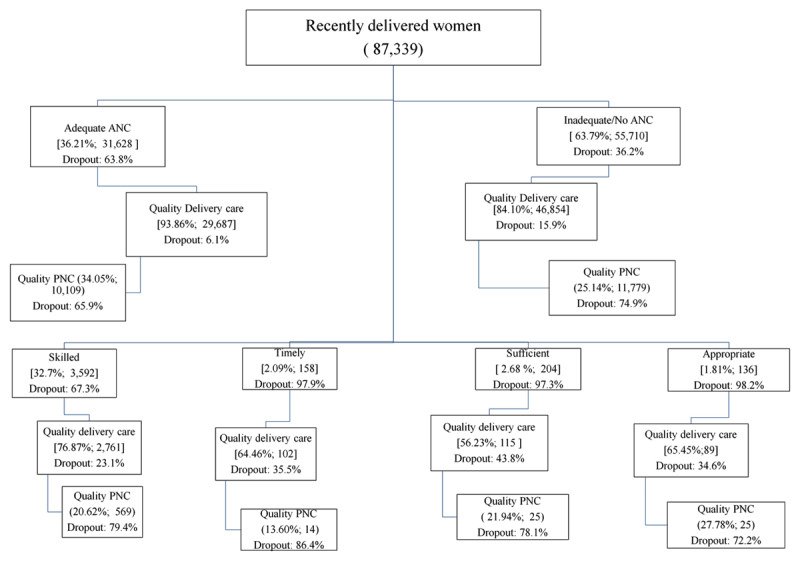
Utilization of quality maternal and newborn healthcare services and its components along the continuum of care in urban India (2015–16).

Out of total 184 641 women who delivered births only 23.5% (43 374) received QANC, and 7.1% dropout was observed for QDC (Figure 1). A significant drop out was observed among the newborns of accessing QPNC for both the newborns of women accessing QANC (64.9%) and either inadequate or no ANC (76.1%). Most of the eligible women accessed exclusive skilled ANC (37.1%; 19,326) compared to other dimensions where more than 70% dropouts along the continuum were noticed for QPNC among newborns. Of the total of 97 302 residing in the nine high focus states (Figure 2), 12.1% (11 745) availed QANC yielding a dropout of 87.9% (Figure 3).

### Disparity across states utilization of quality antenatal, delivery and postnatal care

**Figure 4:**
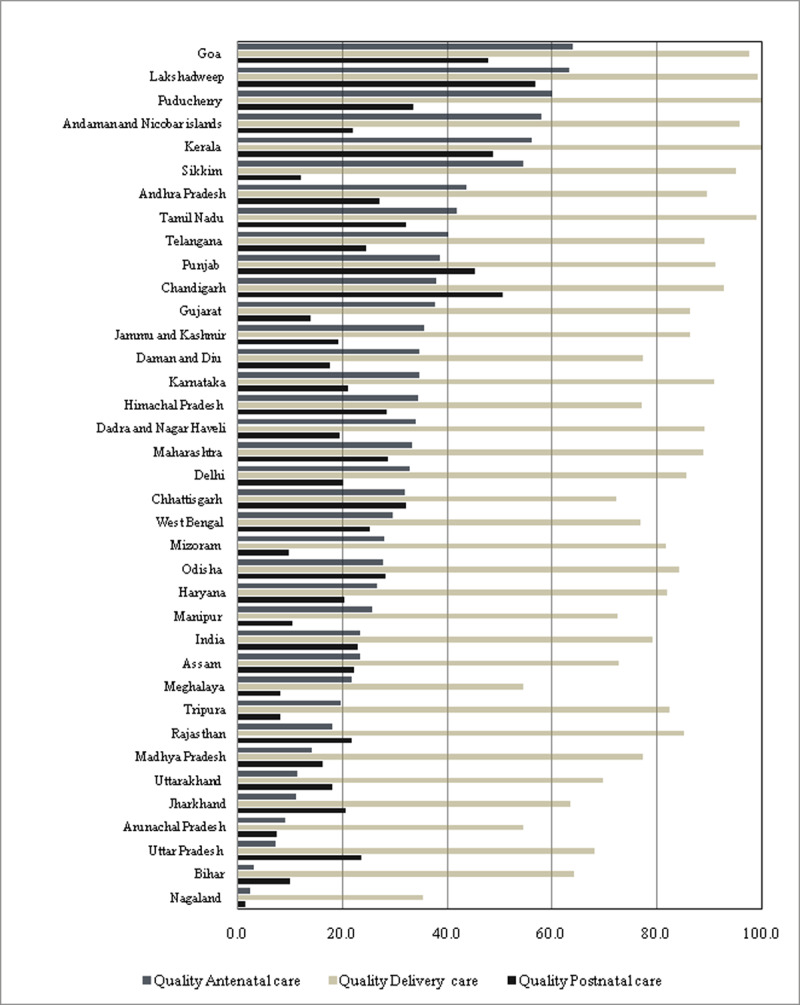
State-wise utilization of quality maternal and newborn healthcare services along the continuum of care (in%).

State-wise pattern clearly suggests that coverage of QDC across states were considerably higher than QANC and QPNC (Figure 4). In Bihar where only 3.1% women received QANC, about 64.2% received QDC. Exception to this pattern were less densely populated states such as Goa and Lakshadweep where gap between services was comparatively lower. The coverage of QANC was below 20% in many bigger states like Bihar (3.1%), Uttar Pradesh (7.2%), Jharkhand (11.3%), Madhya Pradesh (14.1%) and Rajasthan (18.1%). As evident in Odisha and Haryana, given the same level of QANC coverage among states, coverage of QDC and QPNC differed markedly.

Rural areas in Bihar (2.8%, 95% CI: 2.5–3.1), Nagaland (1.4%, 95% CI: 1.0 – 2.1) and Uttar Pradesh (4.6%, 95% CI: 4.3–5.0) were found with least utilization of ANC (Table 1). Least urban-rural disparity for coverage of quality PNC was observed in Andaman and Nicobar Islands, Delhi and West Bengal.

**Table 1:** Urban-rural differentials observed among women with regards to quality Maternal and Newborn care as captured by NFHS-4 (2015–16).


STATES	QUALITY ANC					QUALITY DELIVERY CARE					QUALITY PNC				
		
	*RURAL*		*URBAN*		*U VS R*	*RURAL*		*URBAN*		*U VS R*	*RURAL*		*URBAN*		*U VS R*

Andaman and Nicobar islands	62.5	[57.2,67.6]	50.9	[37.5,64.2]	–18.6	94.4	[90.5,96.8]	98.0	[92.8,99.4]	3.8	21.9	[16.5,28.5]	22.2	[11.5,38.4]	1.4

Andhra Pradesh	43.2	[40.2,46.3]	45.0	[40.7,49.5]	4.2	87.7	[85.2,89.9]	93.8	[91.0,95.8]	7.0	26.1	[23.5,28.9]	30.0	[25.4,35.1]	14.9

Arunachal Pradesh	8.6	[7.2,10.3]	11.0	[8.3,14.5]	27.9	46.2	[42.9,49.5]	82.1	[77.5,85.9]	77.7	7.9	[6.5,9.7]	5.7	[4.0,8.1]	–27.8

Assam	22.0	[20.5,23.6]	34.9	[30.3,39.9]	58.6	70.4	[68.4,72.3]	92.7	[89.6,95.0]	31.7	22.0	[20.4,23.6]	23.8	[19.9,28.2]	8.2

Bihar	2.8	[2.5,3.1]	5.9	[4.5,7.7]	110.7	62.7	[61.4,64.0]	76.3	[73.0,79.4]	21.7	9.4	[8.7,10.2]	16.1	[13.4,19.1]	71.3

Chandigarh	33.3	[33.3,33.3]	38.0	[28.4,48.7]	14.1	100.0	*	92.4	[85.8,96.1]	–7.6	66.7	[66.7,66.7]	49.8	[38.5,61.2]	–25.3

Chhattisgarh	28.9	[26.7,31.3]	42.8	[38.4,47.4]	48.1	68.7	[66.5,70.9]	84.4	[80.8,87.4]	22.9	30.1	[27.9,32.5]	38.9	[34.7,43.3]	29.2

Dadra and Nagar Haveli	46.4	[32.2,61.2]	18.3	[11.4,28.0]	–60.6	85.7	[73.9,92.7]	93.2	[85.6,96.9]	8.8	24.7	[14.9,38.1]	12.7	[5.8,25.7]	–48.6

Daman and Diu	40.4	[27.6,54.6]	32.5	[24.6,41.7]	–19.6	81.9	[69.5,90.0]	75.5	[67.4,82.1]	–7.8	23.5	[14.9,35.2]	15.2	[9.4,23.6]	–35.3

Goa	57.0	[48.4,65.2]	67.9	[59.1,75.6]	19.1	98.6	[95.7,99.5]	97.0	[93.4,98.7]	–1.6	47.2	[37.0,57.7]	48.2	[41.3,55.1]	2.1

Gujarat	31.0	[28.8,33.3]	46.7	[42.1,51.4]	50.6	82.0	[79.9,83.9]	91.9	[89.9,93.5]	12.1	13.4	[11.7,15.4]	14.7	[11.4,18.6]	9.7

Haryana	26.4	[24.1,28.7]	27.3	[24.1,30.7]	3.4	82.4	[80.7,84.0]	80.9	[77.7,83.8]	–1.8	21.3	[19.2,23.6]	18.6	[15.8,21.8]	–12.7

Himachal Pradesh	33.3	[30.7,36.0]	47.6	[37.7,57.8]	42.9	75.7	[72.7,78.5]	91.3	[84.4,95.3]	20.6	27.8	[25.0,30.8]	35.9	[27.0,46.0]	29.1

Jammu and Kashmir	33.4	[31.1,35.7]	42.2	[36.5,48.1]	26.3	82.5	[80.7,84.1]	97.4	[95.9,98.3]	18.1	17.1	[15.5,18.8]	25.9	[20.4,32.2]	51.5

Jharkhand	9.4	[8.4,10.5]	18.6	[16.0,21.7]	97.9	58.7	[56.8,60.5]	83.2	[80.6,85.4]	41.7	20.1	[18.5,21.8]	23.2	[20.1,26.7]	15.4

Karnataka	35.0	[32.1,38.0]	34.5	[29.6,39.8]	–1.4	91.2	[89.9,92.3]	90.8	[87.3,93.4]	–0.4	21.8	[19.7,24.0]	20.0	[16.2,24.4]	–8.3

Kerala	55.5	[51.9,59.0]	56.7	[52.1,61.2]	2.2	100.0	[99.9,100.0]	99.8	[99.1,99.9]	–0.2	49.8	[45.7,54.0]	47.4	[42.8,51.9]	–4.8

Lakshadweep	61.8	[40.6,79.2]	63.5	[54.9,71.4]	2.8	94.6	[69.6,99.3]	100.0		5.7	60.6	[54.5,66.3]	56.2	[48.5,63.5]	–7.3

Madhya Pradesh	10.7	[9.9,11.5]	23.1	[20.7,25.8]	115.9	72.5	[71.2,73.7]	89.9	[88.2,91.3]	24.0	15.6	[14.5,16.6]	18.2	[16.4,20.2]	16.7

Maharashtra	31.2	[29.0,33.4]	35.7	[31.5,40.1]	14.4	84.7	[82.7,86.5]	93.8	[92.2,95.1]	10.7	29.2	[27.2,31.3]	28.4	[24.5,32.6]	–2.7

Manipur	22.1	[19.6,24.7]	32.1	[27.7,36.8]	45.2	64.3	[61.3,67.1]	87.3	[84.5,89.7]	35.8	8.3	[6.7,10.3]	14.3	[11.5,17.7]	72.3

Meghalaya	19.3	[16.8,22.1]	35.3	[29.2,41.9]	82.9	48.6	[44.5,52.7]	89.7	[85.7,92.6]	84.6	7.3	[5.8,9.1]	14.5	[9.9,20.8]	98.6

Mizoram	18.8	[15.6,22.4]	36.1	[32.4,39.9]	92.0	62.8	[58.0,67.4]	98.0	[97.1,98.6]	56.1	6.8	[4.8,9.6]	12.6	[9.9,16.0]	85.3

Nagaland	1.4	[1.0,2.1]	4.9	[3.6,6.6]	250.0	26.5	[23.9,29.3]	56.8	[52.3,61.1]	114.3	1.2	[0.8,1.8]	2.1	[1.1,4.0]	75.0

Delhi	29.7	[22.6,38.0]	32.8	[29.0,36.7]	10.4	80.2	[57.4,92.4]	85.8	[82.5,88.5]	7.0	19.8	[7.6,42.6]	20.1	[15.6,25.6]	1.5

Odisha	26.8	[25.4,28.3]	32.6	[28.6,36.8]	21.6	83.6	[82.2,84.8]	88.2	[81.1,92.8]	5.5	29.0	[27.5,30.6]	23.7	[20.2,27.5]	–18.3

Puducherry	46.4	[36.8,56.3]	65.5	[55.8,74.1]	41.2	100.0		99.8	[99.2,100.0]	–0.2	36.8	[28.7,45.8]	32.1	[22.6,43.4]	–12.8

Punjab	37.5	[34.5,40.6]	40.5	[36.5,44.6]	8.0	92.0	[90.5,93.3]	89.8	[85.5,93.0]	–2.4	45.8	[42.7,49.0]	44.7	[39.9,49.6]	–2.4

Rajasthan	15.2	[14.1,16.4]	28.2	[25.7,30.7]	85.5	83.3	[82.1,84.5]	91.2	[89.7,92.5]	9.5	21.2	[19.8,22.7]	23.7	[21.2,26.5]	11.8

Sikkim	52.9	[48.2,57.6]	57.3	[49.7,64.6]	8.3	94.8	[92.2,96.6]	95.4	[91.3,97.6]	0.6	13.5	[9.7,18.4]	9.5	[5.5,15.7]	–29.6

Tamil Nadu	40.9	[38.2,43.6]	42.9	[39.5,46.3]	4.9	98.7	[97.8,99.2]	99.1	[98.6,99.5]	0.4	32.5	[30.1,35.0]	31.8	[29.1,34.6]	–2.2

Tripura	17.0	[13.9,20.7]	27.4	[21.2,34.7]	61.2	78.6	[75.1,81.8]	93.1	[87.7,96.2]	18.4	8.6	[6.2,11.6]	6.9	[3.8,12.1]	–19.8

Uttar Pradesh	4.6	[4.3,5.0]	16.8	[15.4,18.3]	265.2	66.9	[65.9,67.8]	73.1	[71.3,74.7]	9.3	21.5	[20.6,22.4]	31.6	[29.6,33.8]	47.0

Uttarakhand	9.2	[7.9,10.7]	16.1	[13.1,19.7]	75.0	64.7	[62.1,67.3]	79.8	[76.0,83.1]	23.3	15.8	[13.9,17.8]	22.9	[18.9,27.4]	44.9

West Bengal	28.4	[26.1,30.9]	32.4	[28.3,36.9]	14.1	73.5	[70.8,76.0]	85.0	[79.7,89.1]	15.6	25.1	[23.0,27.3]	25.3	[21.5,29.5]	0.8

Telangana	37.9	[34.5,41.4]	42.7	[37.1,48.6]	12.7	84.8	[82.2,87.0]	93.8	[91.5,95.6]	10.6	27.7	[24.5,31.0]	21.2	[16.9,26.3]	–23.5

Total	19.5	[19.1,19.9]	32.9	[31.9,33.9]	68.7	75.2	[74.8,75.6]	88.5	[87.9,89.1]	17.7	21.8	[21.4,22.2]	26.1	[25.2,27.0]	19.7


* Small sample size obtained from Chandigarh (n = 6).

#### District-wise disparity in coverage of quality ANC, delivery and PNC

**Figure 5:**
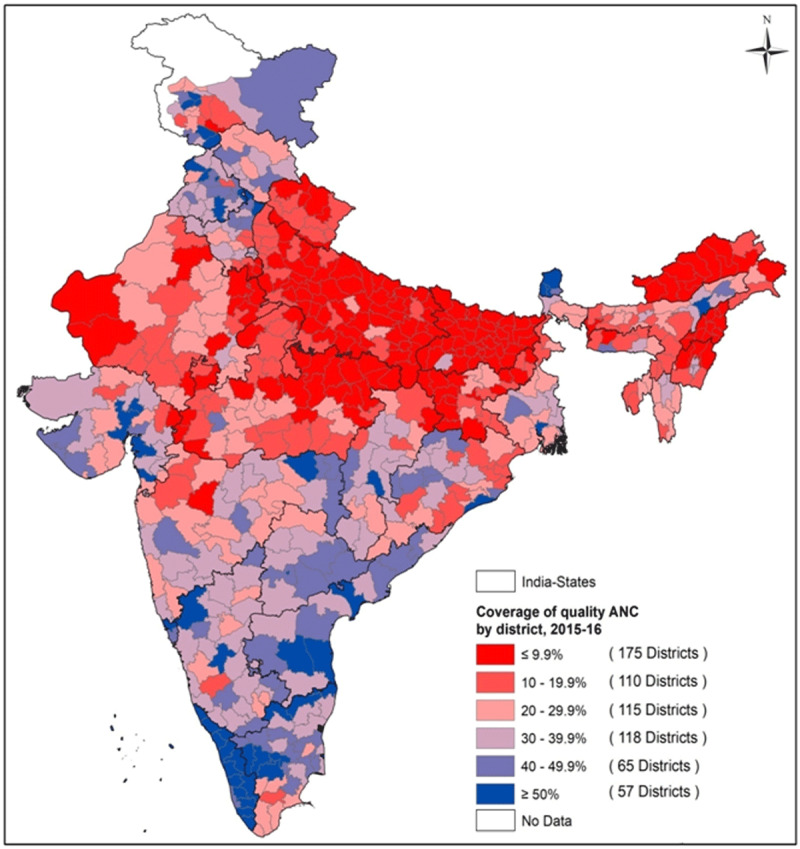
Spatial variation for utilization of quality ANC across 640 districts of India, 2015–16.

**Figure 6:**
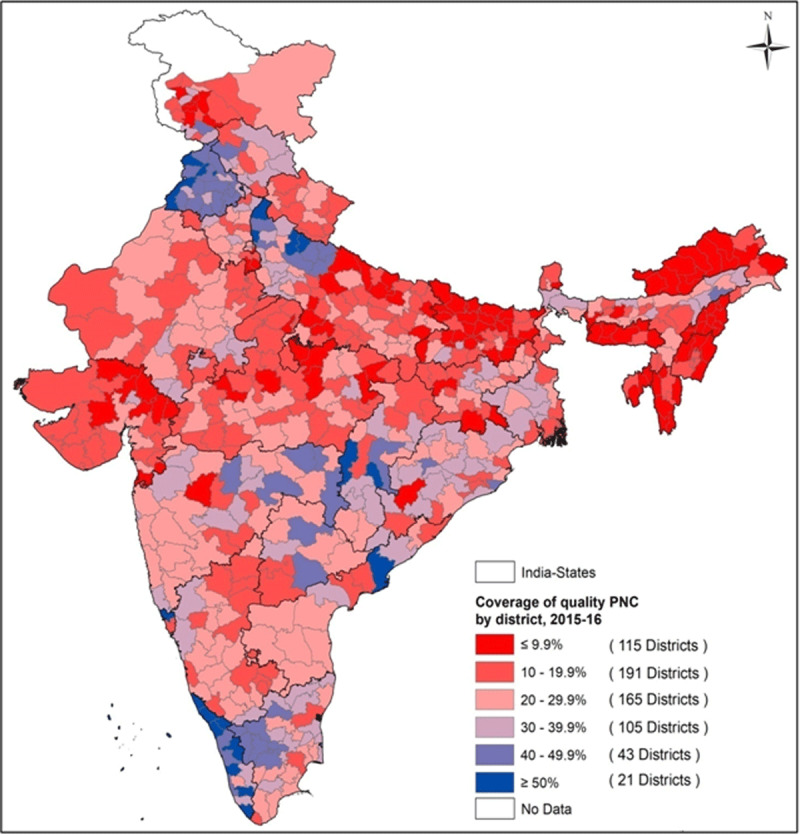
Spatial variation for utilization of quality delivery care across 640 districts of India, 2015–16.

**Figure 7:**
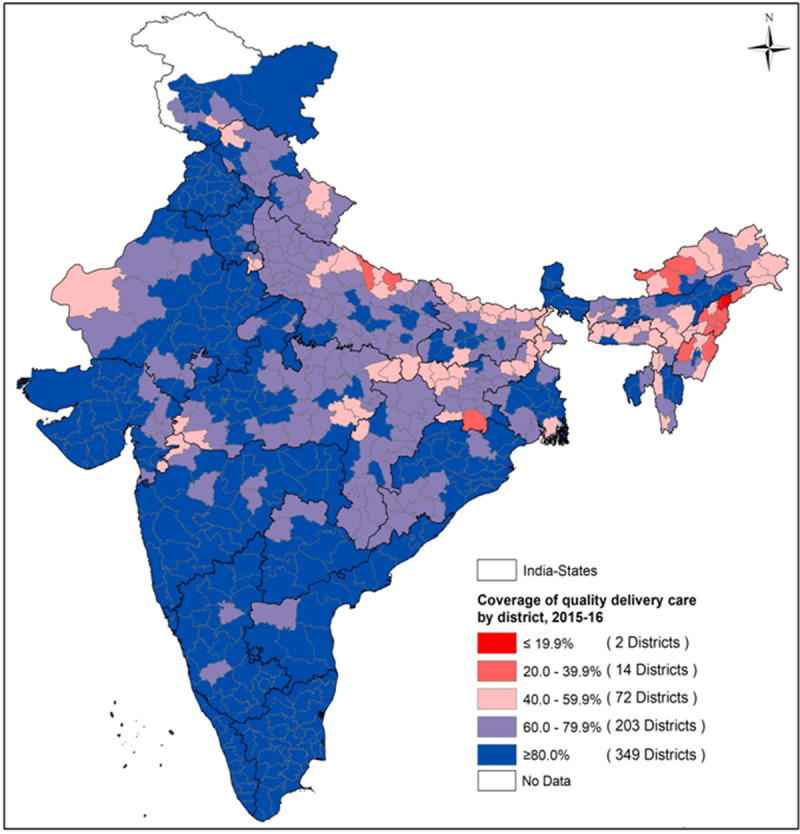
Spatial variation for utilization of quality PNC across 640 districts of India, 2015–16.

Figure 5 displays the coverage of QANC across 640 districts of India where 175 district were observed with less than 10% coverage and were concentrated mostly among Uttar Pradesh, Uttarakhand, Bihar, Arunachal Pradesh, Nagaland, Jharkhand and parts of Madhya Pradesh and Rajasthan. Most districts in Tamil Nadu, Andhra Pradesh, Telangana, Kerala, Punjab and parts of Gujarat and Haryana had more than 30% coverage of QANC. Most districts were found with more than 60% coverage of QDC (Figure 6) while less than 20% coverage were noted in districts of Arunachal Pradesh, Nagaland, Uttar Pradesh and Jharkhand. Figure 7 presented a grim scenario of QPNC for newborns where only 21 (out of 640) districts were noted to account for more than 50% coverage among the eligible demographic, most of which were concentrated in Kerala and Punjab.

### Socioeconomic disparity in utilization of quality ANC, delivery and PNC

The coverage of QANC was found to be significantly associated women’s education (9.9% among illiterate to 37% among women with more than 12 years of education) and advancement of economic status of the household (7.9% among the poorest household to 40.4% among the richest household) (Table 2). Similar pattern was observed for coverage of QDC and QPNC. More than 50% women across all the socioeconomic factors received quality delivery care (Table 3). Overall coverage of QPNC was relatively low across all the socioeconomic factors, however, region-wise, highest coverage was noted in the southern region (29.3%, 95% CI: 28.2–30.4) and least in the Northeastern region (18.1%, 95%CI: 17.1–19.2) (Table 4). Of the three factors difference in coverage between the high focus states and non-high focus states was noted the highest for QANC (difference of 24.1%) followed by QDC (16% difference) and QPNC (6.4% difference).

**Table 2:** Socioeconomic differentials observed among women with regards to quality ANC accessed as captured by NFHS-4 (2015–16).


BACKGROUND VARIABLES	NO ANC		INADEQUATE ANC		ADEQUATE ANC	
		
	%	95% CI	%	95% CI	%	95% CI

**Mother’s age at child birth**	χ^2^ = 138.1; p = <0.01					

less than 19 (n = 13,849)	15.4	[14.6,16.3]	62.1	[60.8,63.3]	22.5	[21.4,23.6]

20–24 (n = 75,353)	15.1	[14.7,15.5]	60.4	[59.7,61.0]	24.6	[24.0,25.2]

25–29 (n = 62,536)	17.5	[17.1,18.0]	58.2	[57.6,58.9]	24.3	[23.7,24.8]

30–34 (n = 26,772)	22.0	[21.2,22.7]	55.9	[54.9,56.8]	22.2	[21.3,23.1]

35–39 (n = 9,412)	30.2	[28.8,31.6]	53.8	[52.2,55.4]	15.9	[14.7,17.2]

40–49 (n = 2,976)	44.6	[42.1,47.1]	44.9	[42.5,47.4]	10.5	[8.9,12.3]

**Women’s education**	χ^2^ = 902.3; p = <0.01					

no schooling (n = 55,460)	34.8	[34.2,35.4]	55.3	[54.7,55.9]	9.9	[9.5,10.3]

less than 5 years (n = 11,701)	19.4	[18.4,20.5]	62.5	[61.1,63.9]	18.1	[16.9,19.3]

5–7 (n = 29,971)	14.6	[14.0,15.2]	63.1	[62.2,64.0]	22.3	[21.5,23.2]

8–9 (n = 34,576)	12.7	[12.1,13.2]	62.3	[61.4,63.2]	25	[24.2,25.9]

10–11 (n = 22,124)	8.9	[8.3,9.5]	58.4	[57.3,59.5]	32.7	[31.7,33.8]

12 or more years (n = 37,066)	6.4	[6.0,6.8]	56.6	[55.7,57.4]	37	[36.2,37.9]

**Parity**	χ^2^ = 1002.6; p = <0.01					

1 (n = 61,807)	10.6	[10.2,10.9]	59.3	[58.6,60.0]	30.1	[29.5,30.8]

2 to 3 (n = 95,548)	16.7	[16.3,17.1]	59.6	[59.1,60.2]	23.7	[23.2,24.2]

4 to 5 (n = 24,879)	33.1	[32.3,34.0]	57.1	[56.2,58.0]	9.7	[9.1,10.3]

6 or more (n = 8,664)	47.3	[45.9,48.8]	48.5	[47.0,49.9]	4.2	[3.6,4.9]

**Economic Status**	χ^2^ = 1131.5; p = <0.01					

Poorest (n = 46,782)	37.7	[37.0,38.5]	54.4	[53.6,55.1]	7.9	[7.5,8.3]

Poorer (n = 43,739)	19.5	[18.9,20.1]	63.2	[62.5,64.0]	17.3	[16.7,17.9]

Middle (n = 38,393)	11.9	[11.4,12.4]	61.9	[61.1,62.7]	26.2	[25.4,27.0]

Richer (n = 33,212)	8.2	[7.7,8.7]	59.8	[58.8,60.8]	32	[31.0,33.0]

Richest (n = 28,772)	5.1	[4.6,5.7]	54.5	[53.4,55.6]	40.4	[39.3,41.5]

**Religion**	χ^2^ = 48.7; p = <0.01					

Hindu (n = 138,343)	17.6	[17.3,18.0]	58.9	[58.4,59.4]	23.5	[23.1,23.9]

Muslim (n = 29,309)	20.3	[19.4,21.3]	58.8	[57.7,59.9]	20.9	[19.9,21.9]

Others (n = 23,246)	10.9	[9.8,12.0]	57.3	[55.6,59.1]	31.8	[30.1,33.5]

**Caste**	χ^2^ = 66.4; p = <0.01					

Others (n = 34,705)	12	[11.4,12.6]	58.9	[57.9,59.8]	29.1	[28.2,30.1]

SC (n = 35,170)	19.3	[18.6,19.9]	59.1	[58.2,60.0]	21.6	[20.8,22.5]

ST (n = 37,889)	21.6	[20.7,22.6]	59.4	[58.3,60.5]	19	[18.1,19.9]

OBC (n = 74,060)	19	[18.5,19.4]	58.1	[57.5,58.6]	23	[22.5,23.5]

**Place of residence**	χ^2^ = 596.9; p = <0.01					

Rural (n = 143,065)	21.1	[20.8,21.5]	59.3	[58.9,59.8]	19.5	[19.1,19.9]

Urban (n = 47,833)	9.6	[9.0,10.2]	57.5	[56.5,58.5]	32.9	[31.9,33.9]

**Region**	χ^2^ = 500.2; p = <0.01							

South (n = 19,907)	5.8	[5.2,6.5]	52.2	[51.0,53.4]	42	[40.8,43.2]

North (n = 36,079)	13.1	[12.4,13.8]	61.6	[60.8,62.5]	25.3	[24.5,26.1]

Central (n = 52,952)	23.4	[22.8,24.0]	65.3	[64.7,65.9]	11.3	[10.9,11.8]

East (n = 39,243)	27.7	[26.9,28.4]	56.8	[56.0,57.7]	15.5	[14.8,16.2]

Northeast (n = 28,825)	14	[13.0,15.0]	63.6	[62.4,64.7]	22.5	[21.4,23.6]

West (n = 13,892)	10	[9.2,10.9]	55	[53.3,56.8]	35	[33.3,36.7]

**High focus states of India**	χ^2^ = 2579.0; p = <0.01					

Non-High focus states (n = 78,380)	8.5	[8.0,8.9]	55.3	[54.5,56.1]	36.2	[35.4,37.0]

High focus states (n = 112,518)	26	[25.6,26.4]	61.9	[61.5,62.4]	12.1	[11.8,12.4]

**At least 4 ANC visits**	χ^2^ = 139.4; p = 0.000					

Less than 4 (n = 101,460)	36.3	[35.7,36.9]	63.7	[63.1,64.3]	0	

Four or more (n = 89,438)	0		54.1	[53.5,54.8]	45.9	[45.2,46.5]

**Intake of IFA 100+**	χ^2^ = 4016.9; p = <0.01					

No (n = 138,082)	22.9	[22.5,23.3]	64.2	[63.7,64.6]	13	[12.6,13.3]

Yes (n = 52,816)	5.9	[5.5,6.3]	46.4	[45.7,47.2]	47.7	[46.9,48.5]

**Place of delivery**	χ^2^ = 381.1; p = <0.01					

Respondent’s home (n = 37,758)	41.8	[40.9,42.7]	51.5	[50.7,52.4]	6.7	[6.2,7.2]

Other’s home (n = 381)	35.1	[28.0,43.0]	52.2	[44.5,59.8]	12.7	[8.5,18.6]

Parents’ home (n = 4,013)	35	[32.8,37.2]	53.6	[51.4,55.9]	11.4	[9.9,13.2]

Public: govt./munic. hospital (n = 47,772)	11.1	[10.6,11.6]	60.1	[59.2,61.0]	28.8	[28.0,29.6]

Public: govt. dispensary (n = 3,152)	14.8	[13.0,16.8]	62.6	[60.0,65.2]	22.5	[20.3,24.9]

Public: UHC/UHP/UFWC(n = 2,919)	17.6	[15.6,19.9]	54.4	[51.8,57.0]	27.9	[25.5,30.6]

Public: CHC/rural hospital/block PHC (n = 35,265)	18.8	[18.2,19.5]	65.3	[64.5,66.0]	15.9	[15.3,16.5]

Public: phc/additional PHC(n = 13,896)	16.5	[15.5,17.5]	62.2	[60.9,63.6]	21.3	[20.2,22.4]

Public: sub-centre (n = 2,373)	15.7	[13.7,17.9]	65.2	[62.1,68.1]	19.2	[16.5,22.1]

Other public sector health facility (n = 238)	6.6	[3.8,11.1]	63.3	[54.2,71.5]	30.1	[22.5,39.0]

Private: hospital/maternity home/clinic (n = 40,701)	7.9	[7.5,8.4]	57.4	[56.7,58.2]	34.7	[33.9,35.5]

Other private sector health facility (n = 942)	14.4	[11.6,17.7]	64.7	[60.3,68.8]	20.9	[17.5,24.8]

NGO or Trust hospital/clinic (n = 927)	6.6	[4.7,9.2]	52.7	[47.4,58.0]	40.7	[35.2,46.3]

other (n = 460)	22.5	[17.7,28.2]	58.4	[51.9,64.6]	19.1	[14.1,25.2]

**Skilled PNC on day 1**	χ^2^ = 1262.8; p = <0.01					

No skilled PNC on day 1 (n = 150,400)	21.2	[20.8,21.6]	58.6	[58.1,59.1]	20.2	[19.8,20.6]

Skilled PNC on day 1 (n = 40,498)	6	[5.7,6.4]	59.4	[58.6,60.3]	34.5	[33.7,35.4]


**Table 3:** Socioeconomic differentials observed among women with regards to quality delivery care accessed as captured by NFHS-4 (2015 16).


BACKGROUND VARIABLES	NO QUALITY DELIVERY CARE		QUALITY DELIVERY CARE	
	
	%	95% CI	%	95% CI

**Mother’s age at child birth**	χ^2^ = 240.3; p = <0.01				

less than 19 (n = 13,849)	18.3	[17.4,19.3]	81.7	[80.7,82.6]

20–24 (n = 75,353)	18.5	[18.0,18.9]	81.5	[81.1,82.0]

25–29 (n = 62,536)	20.6	[20.1,21.1]	79.4	[78.9,79.9]

30–34 (n = 26,772)	24.2	[23.4,25.0]	75.8	[75.0,76.6]

35–39 (n = 9,412)	33.5	[32.1,34.9]	66.5	[65.1,67.9]

40–49 (n = 2,976)	48.5	[45.9,51.0]	51.5	[49.0,54.1]

**Women’s education**	χ^2^ = 1663.1 ; p = <0.01				

no schooling (n = 55,460)	38.5	[37.9,39.2]	61.5	[60.8,62.1]

less than 5 years (n = 11,701)	29.9	[28.5,31.3]	70.1	[68.7,71.5]

5–7 (n = 29,971)	20.7	[19.9,21.4]	79.3	[78.6,80.1]

8–9 (n = 34,576)	15.3	[14.7,15.8]	84.7	[84.2,85.3]

10–11 (n = 22,124)	9.7	[9.2,10.3]	90.3	[89.7,90.8]

12 or more years (n = 37,066)	6.2	[5.9,6.6]	93.8	[93.4,94.1]

**Parity**	χ^2^ = 2233.3 ; p = <0.01				

1 (n = 61,807)	10.9	[10.5,11.3]	89.1	[88.7,89.5]

2 to 3 (n = 95,548)	20.9	[20.5,21.3]	79.1	[78.7,79.5]

4 to 5 (n = 24,879)	39.1	[38.2,40.0]	60.9	[60.0,61.8]

6 or more (n = 8,664)	51.8	[50.3,53.3]	48.2	[46.7,49.7]

**Economic Status**	χ^2^ = 1904.6 ; p = <0.01				

Poorest (n = 46,782)	41	[40.2,41.7]	59	[58.3,59.8]

Poorer (n = 43,739)	25.3	[24.6,26.0]	74.7	[74.0,75.4]

Middle (n = 38,393)	15.4	[14.9,16.0]	84.6	[84.0,85.1]

Richer (n = 33,212)	10.1	[9.5,10.7]	89.9	[89.3,90.5]

Richest (n = 28,772)	5.5	[5.1,6.0]	94.5	[94.0,94.9]

**Religion**	χ^2^ = 202.2; p = <0.01				

Hindu (n = 138,343)	19.2	[18.8,19.5]	80.8	[80.5,81.2]

Muslim (n = 29,309)	29.7	[28.5,31.0]	70.3	[69.0,71.5]

Others (n = 23,246)	18.1	[16.6,19.6]	81.9	[80.4,83.4]

**Caste**	χ^2^ = 140.5; p = <0.01				

Others (n = 34,705)	15.8	[15.0,16.6]	84.2	[83.4,85.0]

SC (n = 35,170)	21.7	[21.0,22.4]	78.3	[77.6,79.0]

ST (n = 37,889)	32.2	[31.1,33.4]	67.8	[66.6,68.9]

OBC (n = 74,060)	19.9	[19.4,20.4]	80.1	[79.6,80.6]

**Place of residence**	χ^2^ = 833.1; p = <0.01				

Rural (n = 143,065)	24.8	[24.4,25.2]	75.2	[74.8,75.6]

Urban (n = 47,833)	11.5	[10.9,12.1]	88.5	[87.9,89.1]

**Region**	χ^2^ = 592.6; p = <0.01				

South (n = 19,907)	6.1	[5.5,6.6]	93.9	[93.4,94.5]

North (n = 36,079)	15.7	[15.1,16.4]	84.3	[83.6,84.9]

Central (n = 52,952)	29.1	[28.5,29.7]	70.9	[70.3,71.5]

East (n = 39,243)	29.3	[28.4,30.2]	70.7	[69.8,71.6]

Northeast (n = 28,825)	29.1	[27.8,30.5]	70.9	[69.5,72.2]

West (n = 13,892)	12	[11.1,13.0]	88	[87.0,88.9]

**High focus states of India**	χ^2^ = 1442.0; p = <0.01				

Non-High focus states (n = 78,380)	12.4	[11.8,12.9]	87.6	[87.1,88.2]

High focus states (n = 112,518)	28.4	[28.0,28.9]	71.6	[71.1,72.0]

**At least 4 ANC visits**	χ^2^ = 4033.1 ; p = <0.01				

Less than 4 (n = 101,460)	31.9	[31.3,32.4]	68.1	[67.6,68.7]

Four or more (n = 89,438)	10.3	[10.0,10.7]	89.7	[89.3,90.0]

**Intake of IFA 100+**	χ^2^ = 2279.1 ; p = <0.01				

No (n = 138,082)	25.6	[25.2,26.1]	74.4	[73.9,74.8]

Yes (n = 52,816)	9.7	[9.3,10.2]	90.3	[89.8,90.7]

**Place of delivery**	χ^2^ = 5641.4; p = <0.01				

Respondent’s home (n = 37,758)	100		0	

Other’s home (n = 381)	100		0	

Parents’ home (n = 4,013)	100		0	

Public: govt./munic. hospital (n = 47,772)	1.9	[1.8,2.1]	98.1	[97.9,98.2]

Public: govt. dispensary (n = 3,152)	3.9	[3.0,5.1]	96.1	[94.9,97.0]

Public: UHC/UHP/UFWC(n = 2,919)	3	[2.3,3.8]	97	[96.2,97.7]

Public: CHC/rural hospital/block PHC (n = 35,265)	2.7	[2.5,3.0]	97.3	[97.0,97.5]

Public: phc/additional PHC(n = 13,896)	2.6	[2.2,3.0]	97.4	[97.0,97.8]

Public: sub-centre (n = 2,373)	3.5	[2.5,5.0]	96.5	[95.0,97.5]

Other public sector health facility (n = 238)	2.3	[0.9,5.3]	97.7	[94.7,99.1]

Private: hospital/maternity home/clinic (n = 40,701)	2.3	[2.1,2.6]	97.7	[97.4,97.9]

Other private sector health facility (n = 942)	4.2	[2.9,6.1]	95.8	[93.9,97.1]

NGO or Trust hospital/clinic (n = 927)	4.3	[2.7,6.7]	95.7	[93.3,97.3]

other (n = 460)	100		0	

**Skilled PNC on day 1**	χ^2^ = 2771.4; p = <0.01				

No skilled PNC on day 1 (n = 150,400)	24.9	[24.5,25.4]	75.1	[74.6,75.5]

Skilled PNC on day 1 (n = 40,498)	7.2	[6.8,7.5]	92.8	[92.5,93.2]


**Table 4:** Socioeconomic differentials observed among women with regards to quality PNC accessed as captured by NFHS-4 (2015–16).


BACKGROUND CHARACTERISTICS	NO SKILLED PNC BY DAY 1		SKILLED PNC BY DAY 1	
	
	ROW %	95% CI	ROW %	95% CI

**Mother’s age at child birth**	χ^2^ = 10.8; p = <0.01				

less than 19 (n = 13,849)	76.8	[75.7,78.0]	23.2	[22.0,24.3]

20–24 (n = 75,353)	76.4	[75.9,77.0]	23.6	[23.0,24.1]

25–29 (n = 62,536)	76.7	[76.2,77.3]	23.3	[22.7,23.8]

30–34 (n = 26,772)	77.8	[76.9,78.6]	22.2	[21.4,23.1]

35–39 (n = 9,412)	79.8	[78.2,81.3]	20.2	[18.7,21.8]

40–49 (n = 2,976)	84.4	[82.5,86.2]	15.6	[13.8,17.5]

**Women’s education**	χ^2^ = 161.8; p = <0.01				

no schooling (n = 55,460)	83.4	[82.9,83.9]	16.6	[16.1,17.1]

less than 5 years (n = 11,701)	78.3	[77.1,79.5]	21.7	[20.5,22.9]

5–7 (n = 29,971)	77.1	[76.3,77.9]	22.9	[22.1,23.7]

8–9 (n = 34,576)	75.1	[74.3,75.9]	24.9	[24.1,25.7]

10–11 (n = 22,124)	72.9	[71.8,73.9]	27.1	[26.1,28.2]

12 or more years (n = 37,066)	71.9	[71.1,72.7]	28.1	[27.3,28.9]

**Parity**	χ^2^ = 152.0; p = <0.01				

1 (n = 61,807)	74.4	[73.8,75.0]	25.6	[25.0,26.2]

2 to 3 (n = 95,548)	76.8	[76.2,77.3]	23.2	[22.7,23.8]

4 to 5 (n = 24,879)	82.6	[81.9,83.3]	17.4	[16.7,18.1]

6 or more (n = 8,664)	85.3	[84.2,86.3]	14.7	[13.7,15.8]

**Economic status**	χ^2^ = 261.7; p = <0.01				

Poorest (n = 46,782)	84.8	[84.3,85.3]	15.2	[14.7,15.7]

Poorer (n = 43,739)	79.1	[78.4,79.7]	20.9	[20.3,21.6]

Middle (n = 38,393)	75.4	[74.6,76.1]	24.6	[23.9,25.4]

Richer (n = 33,212)	72.2	[71.3,73.2]	27.8	[26.8,28.7]

Richest (n = 28,772)	70.5	[69.5,71.5]	29.5	[28.5,30.5]

**Religion**	χ^2^ = 29.3; p = 0.000				

Hindu (n = 138,343)	76.9	[76.5,77.3]	23.1	[22.7,23.5]

Muslim (n = 29,309)	78.8	[77.8,79.8]	21.2	[20.2,22.2]

Others (n = 23,246)	71.6	[70.1,73.1]	28.4	[26.9,29.9]

**Caste**	χ^2^ = 10.6; p = <0.01				

Others (n = 34,705)	75.6	[74.7,76.5]	24.4	[23.5,25.3]

SC (n = 35,170)	76.5	[75.7,77.3]	23.5	[22.7,24.3]

ST (n = 37,889)	79.4	[78.4,80.4]	20.6	[19.6,21.6]

OBC (n = 74,060)	76.9	[76.4,77.4]	23.1	[22.6,23.6]

**Place of residence**	χ^2^ = 76.8; p = <0.01				

Rural (n = 143,065)	78.2	[77.8,78.6]	21.8	[21.4,22.2]

Urban (n = 47,833)	73.9	[73.0,74.8]	26.1	[25.2,27.0]

**Region**	χ^2^ = 72.6; p = <0.01				

South (n = 19,907)	70.7	[69.6,71.8]	29.3	[28.2,30.4]

North (n = 36,079)	75.5	[74.6,76.3]	24.5	[23.7,25.4]

Central (n = 52,952)	77.4	[76.8,78.0]	22.6	[22.0,23.2]

East (n = 39,243)	81.6	[80.9,82.3]	18.4	[17.7,19.1]

Northeast (n = 28,825)	81.9	[80.8,82.9]	18.1	[17.1,19.2]

West (n = 13,892)	76.1	[74.5,77.6]	23.9	[22.4,25.5]

**High focus states of India**	χ^2^ = 257.5; p = <0.01				

Non-High focus states (n = 78,380)	73.6	[72.9,74.3]	26.4	[25.7,27.1]

High focus states (n = 112,518)	80	[79.6,80.4]	20	[19.6,20.4]

**At least 4 ANC Visits**	χ^2^ = 1269.6; p = <0.01				

Less than 4 (n = 101,460)	83.8	[83.3,84.2]	16.2	[15.8,16.7]

Four or more (n = 89,438)	70.5	[69.9,71.1]	29.5	[28.9,30.1]

**Intake of IFA 100+**	χ^2^ = 384.1; p = <0.01				

No (n = 138,082)	79.3	[78.9,79.7]	20.7	[20.3,21.1]

Yes (n = 52,816)	71.6	[70.9,72.4]	28.4	[27.6,29.1]

**Place of delivery**	χ^2^ = 197.0; p = <0.01				

Respondent’s home (n = 37,758)	93.1	[92.7,93.5]	6.9	[6.5,7.3]

Other’s home (n = 381)	88.9	[84.6,92.2]	11.1	[7.8,15.4]

Parents’ home (n = 4,013)	91.2	[89.8,92.4]	8.8	[7.6,10.2]

Public: govt./munic. hospital (n = 47,772)	72.8	[71.9,73.6]	27.2	[26.4,28.1]

Public: govt. dispensary (n = 3,152)	75.7	[73.1,78.1]	24.3	[21.9,26.9]

Public: UHC/UHP/UFWC (n = 2,919)	75.3	[72.9,77.6]	24.7	[22.4,27.1]

Public: CHC/Rural hospital/block PHC (n = 35,265)	75.3	[74.5,76.0]	24.7	[24.0,25.5]

Public: PHC/additional PHC (n = 13,896)	75.5	[74.2,76.7]	24.5	[23.3,25.8]

Public: Sub-centre (n = 2,373)	74.5	[71.7,77.1]	25.5	[22.9,28.3]

Other public sector health facility (n = 238)	75.3	[67.2,81.9]	24.7	[18.1,32.8]

Private: hospital/maternity home/clinic (n = 40,701)	71.6	[70.8,72.4]	28.4	[27.6,29.2]

Other private sector health facility (n = 942)	76.6	[72.2,80.4]	23.4	[19.6,27.8]

NGO or trust hospital/clinic (n = 927)	75.4	[70.3,79.8]	24.6	[20.2,29.7]

Other (n = 460)	84.3	[79.7,88.1]	15.7	[11.9,20.3]


### Determinants of quality ANC, delivery and PNC

The logistic regression analysis explained the notable decrease in variance across the two models (Model I: Empty Model; Model II: all the covariates at the individual, household and contextual levels) for the coverage of QANC, QDC and QPNC, however, significant unobserved heterogeneity was present at the district and PSU levels (Table 5).

**Table 5:** Parameter estimates of random effect variances and Intra-class correlation of binary regression conducted for determinants of services along the continuum of care at district and PSU levels, India (2015–16).


PARAMETER ESTIMATES	QUALITY OF ANTENATAL CARE						QUALITY OF DELIVERY CARE						QUALITY OF POSTNATAL CARE FOR NEW BORN					
		
	MODEL 1 (EMPTY MODEL)			MODEL 2 (FULL MODEL)			MODEL 1 (EMPTY MODEL)			MODEL 2 (FULL MODEL)			MODEL 1 (EMPTY MODEL)			MODEL 2 (FULL MODEL)		
					
	SE	P-VALUE	95%CI	SE	P-VALUE	95% CI	SE	P-VALUE	95% CI	SE	P-VALUE	95% CI	SE	P-VALUE	95% CI	SE	P-VALUE	95% CI

**Random-effects Parameters**																		

District level variance	2.01	0.12	1.79–2.26	1.08	0.07	0.95–1.22	1.16	0.11	0.96–1.39	0.79	0.08	0.65–0.97	1.26	0.1	1.08–1.46	1.22	0.1	1.04–1.42

PSU level variance	1.14	0.03	1.09–1.19	1	0.02	0.96–1.05	1.34	0.12	1.13–1.60	1.06	0.11	0.86–0.30	2.66	0.11	2.45–2.90	2.66	0.11	2.45–2.90

**Infraclass correlation coefficient (ICC)**																		

District level	0.31	0.01	0.29–0.34	0.2	0.01	0.18–0.22	0.2	0.01	0.17–0.23	0.15	0.01	0.13–0.18	0.17	0.01	0.15–0.20	0.17	0.01	0.15–0.19

PSU level	0.49	0.01	0.47–0.51	0.39	0.01	0.37–0.40	0.43	0.02	0.40–0.47	0.36	0.02	0.32–0.40	0.54	0.01	0.52–0.56	0.54	0.01	0.52–0.56


Twice the likelihood of QANC utilization were found among the women belonging to the richest wealth quintile (OR: 2.55; 95%CI 2.37–2.75) and received more than 12 years of formal education (OR: 1.95; 95%CI 1.84–2.07) (Table 6). At the contextual level, women residing in the high focus states were less likely to receive QANC (OR: 0.35, 95%CI: 0.30–0.41) than residents of non-high focus states. The likelihood of QDC was lower in poor community (75- 100% of poor in PSU: OR: 0.51, 95%CI: 0.41–0.63). At the contextual level, women residing in high focus states were found with higher odds (OR: 1.41, 95%CI: 1.15–1.74) of availing QPNC for newborns compared to their non-focus states counterparts.

**Table 6:** Binary regression analysis showing determinants of continuum of care, India (2015–16).


BACKGROUND VARIABLES	QUALITY ANTENATAL CARE				QUALITY DELIVERY CARE				QUALITY POSTNATAL CARE			
		
	OR	P-VALUE	95% CI		OR	P-VALUE	95% CI		OR	P-VALUE	95% CI	

***Individual/household level***												

**Age at birth**												

<=19 (ref.)												

20–24	1.12	<0.01	1.06	1.19	1.12	0.24	0.93	1.34	0.99	0.92	0.87	1.13

25–29	1.24	<0.01	1.16	1.32	1.21	0.06	1.0	1.47	0.95	0.46	0.83	1.09

30–34	1.35	<0.01	1.26	1.45	1.71	<0.01	1.36	2.15	0.98	0.76	0.83	1.14

35–39	1.21	<0.01	1.1	1.33	1.51	0.01	1.12	2.03	1.08	0.47	0.87	1.34

40–49	1.31	<0.01	1.12	1.54	1.37	0.17	0.87	2.15	1.1	0.64	0.74	1.61

**Women’s education**												

no schooling (ref.)												

Less than 5 years	1.16	<0.01	1.08	1.25	1	0.97	0.84	1.21	1.44	<0.01	1.2	1.72

5 to 7 years	1.30	<0.01	1.23	1.37	1.14	0.09	0.98	1.31	1.2	0.01	1.05	1.37

8 to 9 years	1.49	<0.01	1.42	1.57	1.43	<0.01	1.22	1.66	1.19	0.01	1.04	1.35

10 to 11 years	1.64	<0.01	1.55	1.74	1.56	<0.01	1.31	1.87	1.1	0.17	0.96	1.26

12 or more years	1.95	<0.01	1.84	2.07	2.06	<0.01	1.71	2.47	1.13	0.08	0.99	1.29

**Parity**												

1 (ref.)												

2 to 3	0.8	<0.01	0.78	0.83	0.47	<0.01	0.42	0.53	1.05	0.19	0.98	1.13

4 to5	0.56	<0.01	0.52	0.59	0.3	<0.01	0.25	0.36	1	0.96	0.85	1.17

6 and above	0.38	<0.01	0.33	0.43	0.23	<0.01	0.17	0.32	1.13	0.52	0.78	1.63

**Gender composition of living children**												

No sons (ref.)												

At least one son	0.99	0.45	0.96	1.02	0.92	0.1	0.83	1.02	1.02	0.59	0.95	1.09

**Wealth quintile**												

Poorest (ref.)												

Poorer	1.37	<0.01	1.3	1.45	1.42	<0.01	1.24	1.64	0.89	0.11	0.77	1.03

Middle	1.67	<0.01	1.58	1.78	1.79	<0.01	1.51	2.11	0.90	0.19	0.78	1.05

Richer	1.98	<0.01	1.85	2.11	2.13	<0.01	1.75	2.59	0.92	0.33	0.79	1.08

Richest	2.55	<0.01	2.37	2.75	2.86	<0.01	2.27	3.61	0.92	0.33	0.77	1.09

**Caste**												

Others (ref.)												

Scheduled Castes	0.9	<0.01	0.86	0.95	0.98	0.81	0.84	1.15	0.98	0.78	0.88	1.1

Scheduled Tribes	0.85	<0.01	0.79	0.9	0.71	<0.01	0.60	0.84	0.89	0.12	0.77	1.03

Other Backward Class	0.93	<0.01	0.89	0.97	0.98	0.83	0.86	1.13	0.99	0.76	0.9	1.08

**Religion**												

Hindu (ref.)												

Muslim	0.97	0.26	0.91	1.02	0.61	<0.01	0.52	0.71	0.98	0.78	0.87	1.11

Others	0.94	0.11	0.88	1.01	0.92	0.37	0.76	1.11	0.96	0.59	0.84	1.11

***Contextual level***												

**Place of current residence**												

Rural (ref.)												

Urban	1.01	0.67	0.96	1.07	1.1	0.18	0.96	1.27	0.91	0.08	0.81	1.01

**High focus states**												

No (ref.)												

Yes	0.35	0	0.3	0.41	0.84	0.08	0.69	1.02	1.41	0	1.15	1.74

**Proportion of illiterate in PSU**												

0–25% (ref.)												

25–50%	0.97	0.32	0.92	1.03	0.82	0.01	0.72	0.95	0.97	0.58	0.87	1.08

50–75%	0.89	<0.01	0.84	0.95	0.71	<0.01	0.61	0.84	0.96	0.57	0.84	1.1

75–100%	0.73	<0.01	0.67	0.8	0.74	0.01	0.61	0.92	0.82	0.06	0.67	1.01

**Proportion of poor in PSU**												

0–25% (ref.)												

25–50%	0.97	0.37	0.91	1.04	0.74	<0.01	0.63	0.87	1.07	0.35	0.93	1.22

50–75%	0.92	0.03	0.86	0.99	0.74	<0.01	0.62	0.89	1.07	0.43	0.91	1.25

75–100%	0.79	<0.01	0.73	0.86	0.51	<0.01	0.41	0.63	1.01	0.92	0.83	1.22


*Note*: OR: Odds Ratio; PSU: Primary Sampling Unit.

## Discussion

This study analyzed nationally representative data to examine the coverage and determinants of quality antenatal, delivery and postnatal care as vital components of continuum of care in India. Results reveal less than a quarter of total eligible women eligible accessing quality antenatal care, out of which majority delivering a healthcare facility under supervision of skilled healthcare personnel. However, a steep decrease in numbers of newborns receiving quality postnatal care. Although, in line with literature utilization of ANC was noted more among the non-high focus states [21], however newborns of women receiving quality ANC and delivery utilizing quality PNC was higher in high focus states. This trend can be explained as a result of recently introduced essential MCH interventions in High Focus States such as establishing Auxiliary Nurse Midwives Training Centres, state quality monitoring units and mother’s aide (Yashoda) services [22]. In comparison to other dimensions of quality ANC, a major proportion of women opting for quality delivery care were found exclusively accessing ANC by a skilled practitioner. Provider competency is known to account for continued utilization of the maternal healthcare services [23]. Feasible models of providing skilled care such as Group ANC as tested recently by Jolivet and team [24] in urban setting of India should be further explored.

National Health Policy has mandated registration of pregnancy and first ANC visit within first trimester making availing benefits of incentive schemes such as Indira Gandhi Matritva Sahyog Yojana (IGMSY) along with management of pregnancy and early detection of complications easier [25]. Less than ten percent of all eligible women availed timely ANC which adds to the evidence pool [26]. Regional customs, age, autonomy and education of the woman have been found to factor in the decision to early register the pregnancy calling for effective community-based education and awareness interventions. Literature has often noted four or more ANC visits and mandated procedures as the proxy for the quality ANC [27,28,29], current study found a very small percentage of women opting for at least four ANC visits or exclusive procedures as a part of a visit. Adding to the evidence pool findings highlights that coverage of service does not necessarily serve as a proxy to the coverage of individual content of the same [30].

Quality delivery care was utilized by majority of the woman who opted for ANC and the same was encouragingly reflected at the district level indicating success of incentivized health policies for institutional births such as Janani Suraksha Yojana reaching the sub-regional levels [31]. High dropouts for quality delivery care was observed among women opting for inadequate or no ANC compared to the adequate ANC seconding the results of the study conducted in rural Mexico [32] where more antenatal care services a woman utilized was found associated with higher chances of skilled birth attendance. In concordance to the individual and household level results, high proportions of illiterate and poor in the community have been found with lower likelihood of accessing skilled birth attendance during delivery. The distinct strength of the study is rooted in using nationally representative data to highlight regional and sub-regional differentials and resulting health inequities related to MCH service access.

The present study finds quality PNC among newborns in the first 24 hours has the least coverage followed by the quality antenatal care across the continuum of care. About 54.5% of total districts in the country were noted with at least 80% coverage of quality delivery care, nearly 70% districts had a lower coverage of both quality ANC and PNC. Findings should aid policymakers in incorporating relevant healthcare interventions at national, regional as well as district levels. However, the cross-sectional nature of the data limits the scope of causality among the variables explored.

## Additional File

The additional file for this article can be found as follows:

10.5334/aogh.3586.s1 Supplementary Table 1. Sample Distribution of women included in the study, India (2015–16).
