# Supplementary material for: Coverage of Quality Maternal and Newborn Healthcare Services in India: Examining Dropouts, Disparity and Determinants
Source: Ann Glob Health. 2022 May 26;88(1):39. doi: 10.5334/aogh.3586 (PMC9138825; doi:10.5334/aogh.3586)
Supplement: Supplementary Table 1. — Sample Distribution of women included in the study, India (2015–16). [file agh-88-1-3586-s1.pdf]

Supplementary Table 1. Sample Distribution of women included in the study, India (2015-16)

Background characteristics	n	%
**Mother's age at child birth**		
Less than 19	13849	7.3
20-24	75353	39.5
25-29	62536	32.8
30-34	26772	14
35-39	9412	4.9
40-49	2976	1.6
**Women's education**		
No schooling	55460	29.1
Less than 5 years	11701	6.1
05-07 years	29971	15.7
08-09 years	34576	18.1
10-11 years	22124	11.6
12 or more years	37066	19.4
**Parity**		
1	61807	32.4
2 to 3	95548	50.1
4 to 5	24879	13
6 or more	8664	4.5
**Economic groups**		
Poorest	46782	24.5
Poorer	43739	22.9
Middle	38393	20.1
Richer	33212	17.4
Richest	28772	15.1
**Religion**		
Hindu	138343	72.5
Muslim	29309	15.4

Others	23246	12.2
**Caste**		
Others	34705	18.2
Scheduled Castes (SCs)	35170	18.4
Scheduled Tribes (STs)	37889	19.8
Other Backward Castes (OBCs)	74060	38.8
**Place of residence**		
Rural	143065	74.9
Urban	47833	25.1
**Region**		
South	19907	10.4
North	36079	18.9
Central	52952	27.7
East	39243	20.6
Northeast	28825	15.1
West	13892	7.3
**High focused states of India**		
Non-High Focused states	78380	41.1
High focused states	112518	58.9
**At least 4 ANC Visits**		
Less than 4	101460	53.1
Four or more	89438	46.9
**Intake of IFA 100+**		
No	138082	72.3
Yes	52816	27.7
**Place of delivery**		
Home	37758	19.8
Other's home	381	0.2
Parents' home	4013	2.1
Public: govt./municipality hospital	47772	25

Public: govt. dispensary	3152	1.7
Public: UHC/UHP/UFWC	2919	1.5
Public: CHC/rural hospital/block phc	35265	18.5
Public: PHC/additional PHC	13896	7.3
Public: sub-centre	2373	1.2
Other public sector health facility	238	0.1
Private: Hospital/maternity home/clinic	40701	21.3
Other private sector health facility	942	0.5
NGO or Trust hospital/clinic	927	0.5
Other	460	0.2
**Skilled PNC on day 1**		
No skilled PNC on day 1	150400	78.8
Skilled PNC on day 1	40498	21.2
**Total**	190898	100

ANC: Antenatal care; UHC: Urban Health Centre; UHP: Urban Health Post; UFWC: Urban Family Welfare Centre; CHC: Community Health Centre; PHC: Primary Health Centre; NGO: Non-government Organization; PNC: Post Natal Care.
